# Commitment Levels of Health Care Providers in Using the District Health Information System and the Associated Factors for Decision Making in Resource-Limited Settings: Cross-sectional Survey Study

**DOI:** 10.2196/23951

**Published:** 2021-03-04

**Authors:** Shuma G Kanfe, Berhanu F Endehabtu, Mohammedjud H Ahmed, Nebyu D Mengestie, Binyam Tilahun

**Affiliations:** 1 Health Informatics Mettu University Metu Zuria Ethiopia; 2 Department of Health Informatics Institute of Public Health University of Gondar Gondar Ethiopia

**Keywords:** commitment, district health information system, decision making, performance monitoring, health facilities, information use

## Abstract

**Background:**

Changing the culture of information use, which is one of the transformation agendas of the Ministry of Health of Ethiopia, cannot become real unless health care providers are committed to using locally collected data for evidence-based decision making. The commitment of health care providers has paramount influence on district health information system 2 (DHIS2) data utilization for decision making. Evidence is limited on health care providers’ level of commitment to using DHIS2 data in Ethiopia. Therefore, this study aims to fill this evidence gap.

**Objective:**

This study aimed to assess the levels of commitment of health care providers and the factors influencing their commitment levels in using DHIS2 data for decision making at public health care facilities in the Ilu Aba Bora zone of the Oromia national regional state, Ethiopia in 2020.

**Methods:**

The cross-sectional quantitative study supplemented by qualitative methods was conducted from February 26, 2020 to April 17, 2020. A total of 264 participants were approached. SPSS version 20 software was used for data entry and analysis. Descriptive and analytical statistics, including bivariable and multivariable analyses, were performed. Thematic analysis was conducted for the qualitative data.

**Results:**

Of the 264 respondents, 121 (45.8%, 95% CI 40.0%-52.8%) respondents showed high commitment levels to use DHIS2 data. The variables associated with the level of commitment to use DHIS2 data were found to be provision of feedback for DHIS2 data use (adjusted odds ratio [AOR] 1.85, 95% CI 1.02-3.33), regular supervision and managerial support (AOR 2.84, 95% CI 1.50-5.37), information use culture (AOR 1.92, 95% CI 1.03-3.59), motivation to use DHIS2 data (AOR 1.80, 95% CI 1.00-3.25), health needs (AOR 3.96, 95% CI 2.11-7.41), and competency in DHIS2 tasks (AOR 2.41, 95% CI 1.27-4.55).

**Conclusions:**

In general, less than half of the study participants showed high commitment levels to use DHIS2 data for decision making in health care. Providing regular supportive supervision and feedback and increasing the motivation and competency of the health care providers in performing DHIS2 data tasks will help in promoting their levels of commitment that can result in the cultural transformation of data use for evidence-based decision making in health care.

## Introduction

Health care providers, in particular, the performance monitoring team (PMT) is a team of multidisciplinary health workforce that is primarily responsible for improving data quality, using information regularly, monitoring the health progress, and improving the performance of health care delivery at all levels of the health care system. PMT members are selected health care providers who are involved in the collection, generation, and utilization of health information for decision making, and they serve as the focal persons in their departments/wards. PMT members in Ethiopia are prominent/selected health care providers who widely participate in using district health information system 2 (DHIS2) data for decision making. The commitment levels of the PMT members to their organizations and the use of health information for decision making is a topical issue that needs some attention in the delivery of quality health care.

Changing the culture of information use at each level of the health system is one of the transformation agendas of the Ministry of Health of Ethiopia. This cannot become real unless health care providers are committed to use locally collected data for evidence-based decision making. Health care providers’ level of commitment to use DHIS2 data could provide comprehensive and dependable information, which is the basis for better decision making [1-3]. This is because DHIS2 data consist of global initiatives by Sustainable Development Goals and Countdown to 2030 that emphasize its contribution to monitoring of service delivery by health care providers [4]. The DHIS2 is used in more than 60 countries, and most global initiatives are interested in using DHIS2 data for monitoring the health performance [5-7]. Facility-based data (DHIS2 data) is one of the major identified strategies to achieve sustainable development goals—especially for maternal mortality and neonatal mortality to reach a global average of only 70 per 100,000 live births by 2030 [2,8].

As per the World Health Organization (WHO), health care providers’ level of commitment has paramount influence on DHIS2 data utilization for decision making that will also be the basis for the provision of quality health service [9]. The WHO and the Institute for Health Metrics and Evaluation have stated that to improve the accuracy and utility of health information for decision making, the commitment levels of health care providers is the base [10]. This is because improving the quality of health service can be affected if health care providers are not responsible in using the highly generated medical data, which is but a mandatory step on the path to reaching the sustainable development goals and universal health coverage [11].

A study in Nigeria has identified that the commitment of health care providers to use health information should be taken into consideration, and currently, the level of commitment among health care providers in Nigeria is 60%-80% [1] A study conducted in Isfahan showed that the compliance of health care providers to use district health information was much lower than WHO standards (90%) and was limited to an average of 35.75% [12]. Another study conducted in Iran at hospitals proposed that health care providers’ level of commitment toward the use of health information was a worthy path that every health care worker needs to be dedicated to in using and implementing routine health information for decision making. Currently, the average score of health care providers’ level of commitment to use and implement routine health information, especially electronic medical records, has been achieved with an average of 74.7% [2]. Another study in Ghana indicated that health care providers are expected to have a sense of promoting responsibility to use health information and should feel committed to improving the health status of the target population. Factors such as punctuality at work, documentation of daily activities, and monitoring of data wisely have been associated with the level of commitment to use information. However, currently, the level of commitment among health care providers, specifically among senior managers, is 77.3% [13].

Studies have identified that health care providers need to be committed to using DHIS2 data, wherein over 90% of the available data have been generated within only 2 years [2,14]. In sub-Saharan Africa, the standard procedure for data use is poor and the measures of the health care performance are very low because of the inadequate commitment of health care providers [15]. The quality of health care depends on the dedication and commitment of health care service providers [2]. Being committed to using DHIS2 data will favor high-quality health systems, thereby ensuring relevant advancements toward achievement of sustainable development goals [2,14]. However, over the years, evidence for low commitment to use data have been less as decisions are not taken based on data [2]. Health information data show inconsistency and poor treatment responses because of the low levels of commitments of health care providers [16,17]. The government’s health facilities are not committed to reporting data on a regular basis, data are not used for setting target programs, and these facilities are unresponsive to timely decision making [2,14]. The WHO has stated that factors that affect the commitment to use DHIS2 data are critical in affecting the quality of health service provision [9,10]. Thus, quality of health care will improve when health managers are committed to the use of health information for decision making because quality in health care is a production of cooperation between the patient and the health care provider in a supportive environment [1]. High-quality routine health system data are highly relevant for monitoring advancement toward achievement of the Millennium Development Goals 4 and 5, which are twins to the Sustainable Development Goal 3. However, the main determinant to reach this stage is the level of commitment toward the utilization of routine health information systems. The evidence for the provision of good-quality health service is lacking due to the low commitment of health care providers toward the utilization of health information systems [2]. Low commitment toward the utilization of DHIS2 data results in the production of late diagnosis and treatment reports and distorts the consistency within data space, making the overall utilization of district health information system for decision making to be low [3].

In Ethiopia, the PMT is one of the major platforms to review the performance, data quality, and information use of the health system at each level. The level of commitment of health care providers (especially PMT members) has direct influence on DHIS2 data utilization for decision making [13,18]. Nevertheless, to the best of our knowledge, evidence is limited on PMT members’ level of commitment to use DHIS2 data and the factors that determine the extent of their commitment levels. Therefore, this study aimed to fill the evidence gap on PMT members’ level of commitment to use DHIS2 data for decision making and the factors that determine their commitment levels.

## Methods

### Study Design and Setting

A quantitative cross-sectional study design supplemented by a qualitative study design was conducted from February 26, 2020 to April 17, 2020. This study was conducted in public health facilities in the Ilu Aba Bora zone, Oromia, Ethiopia. The Ilu Aba Bora zone is one of the zones of the Oromia region of Ethiopia, which is 600 km away from Addis Ababa, Ethiopia. This study covered different types of health facilities, including referral hospitals, primary hospitals, and health care centers located in the southwest region of Ethiopia; 41 health centers and 2 hospitals (1 referral hospital and 1 primary hospital) were assessed as the areas for data collection.

### Study Participants and Sample Size Determination

All selected health care providers who handle data, generate data, and use generated data for their decision making and those who serve as focal persons within their departments, collectively known as the PMT members according to the Ethiopian health system context, were the participants of this study. The total number of study participants within this zone was 264. Each study participant was approached and information was collected. For the qualitative study, purposive sampling techniques were used and the level of saturation was considered and saturated at the seventh participant.

### Ethics Approval and Consent to Participate

This study protocol was reviewed and approved by the ethical review board of the University of Gondar and informed consent was obtained from each study participant. A permission letter was also obtained from each health facility. After the objective of this study was explained, verbal consent was obtained from each participant. The privacy and confidentiality of the information were strictly guaranteed by all data collectors and investigators. The information retrieved was used only for this study. Thus, the names of the participants and other personal identifiers were not included in the data collection tool.

### Operational Definitions

#### PMT Members

The PMT members are the health care providers who serve as the focal persons in their respective departments (health management information system [HMIS] Officer, Medical Director, maternal and child health [MCH] Head, tuberculosis [TB] focal nurse, Triage Head nurse, primary health care unit manager, etc) according to Ethiopian health system contexts and the fact that they are responsible for the generation and utilization of data in addition to their clinical roles.

#### Commitment Level of PMT Members to Use DHIS2 Data

The commitment level of PMT members to use DHIS2 data was measured using 11 questions of the Likert scale, and respondents who scored the median score and higher were categorized as having high level of commitment to use DHIS2 data and those who scored less than the median score were categorized as having low level of commitment to use DHIS2 data.

### Data Collection Tools and Procedures

For the quantitative approach, a self-administered English-version questionnaire was used. For qualitative data, in-depth interviews were conducted using an interview guide and a tape recorder. The maximum and minimum times for the in-depth interviews were 49 minutes and 31 minutes, respectively.

### Data Quality Control

Data were collected by trained data collectors by using questionnaires. Before the actual data collection, a pretest was conducted among 5% of the samples at the Buno Bedele general hospital and health center in the Bedele town. The validity of the questionnaire was determined based on the views of experts and the reliability was obtained by calculating the Cronbach alpha value (α=.82). Qualitative data were collected by an investigator after debriefing an in-depth interview by arranging a favorable time and a place for the interviewee.

### Data Processing and Analysis

The data entry and analysis were performed using SPSS version 20 (IBM Corp). To explain the study population in relation to relevant variables, descriptive statistics was used. Associations between dependent and independent variables were checked and their strengths were presented using odds ratios and 95% confidence intervals. Both bivariable and multivariable logistic regressions were used to assess the associations between the outcomes and explanatory variables. *P* values less than .05 were considered statistically significant in the multivariable logistic regression. The qualitative data were analyzed by thematic analysis methods.

## Results

### Sociodemographic Characteristics of the Study Participants

A total of 264 participants were approached with 100% response rate. About two-thirds of the study participants (186/264, 70.5%) were 30 years of age or younger. The majority of the study participants were from a health center (234/264, 88.6%). More than half of the participants were males (147/264, 55.7%). The majority (203/264, 76.9%) of the study participants had a work experience of 4 years and more. About 156 (59.1%) of the 264 study participants had a bachelor’s degree, whereas only 23 (8.7%) had a master’s degree (Table 1).

**Table 1:** Sociodemographic characteristics of the study participants at the health facilities of Ilu Aba Bora Zone in 2020 (N=264).

Variables, subcategories		Values, n (%)
**Age**		
	≤30 years	186 (70.5)
	>30 years	78 (29.5)
**Sex**		
	Male	147 (55.7)
	Female	117 (44.3)
**Type of facility**		
	Referral hospitals	16 (6.1)
	Primary hospitals	14 (5.3)
	Health center	234 (88.6)
**Educational level**		
	Master’s degree	23 (8.7)
	Bachelor’s degree	156 (59.1)
	Diploma	85 (32.2)
**Work experience**		
	≤3 years	61 (23.1)
	>4 years	203 (76.9)
**Position at facility**		
	Head	101 (38.3)
	Expert	163 (61.7)

### Commitment Level of PMT Members to Use DHIS2 Data for Decision Making

Of the 264 respondents, 121 (45.8%, 95% CI 40.0%-52.8%) had high levels of commitment to use DHIS2 data for decision-making purposes.

### Level of Commitment to Use DHIS2 Data for Decision Making by Sociodemographic Variables

Among 117 female respondents, only 50 (42.7%) had high levels of commitment to use DHIS2 data. Holders of master’s degrees had higher levels of commitment than diploma and degree holders. Those who had more work experience had higher commitment levels to use DHIS2 data than those who had lesser work experience. Respondents serving in the Head positions (60/101, 59.4%) had higher levels of commitment than those serving in the expert positions. This detail is presented in Table 2.

**Table 2:** Commitment levels of the performance monitoring team members to use district health information system in accordance with the sociodemographic characteristics^a^.

Variables		Commitment level to use district health information system 2 data (N=264)	
		Low commitment, n (%)	High commitment, n (%)
**Sex**			
	Female (n=117)	67 (57.3)	50 (42.7)
	Male (n=147)	76 (51.7)	71 (48.3)
**Age**			
	≤30 years (n=186)	96 (51.6)	90 (48.4)
	>30 years (n=78)	47(60.3)	31 (39.7)
**Type of facilities**			
	Referral hospital (n=16)	10 (62.5)	6 (37.5)
	Primary hospitals (n=14)	6 (42.9)	8 (57.1)
	Health center (n=234)	127 (54.3)	107 (45.7)
**Educational level**			
	Master’s degree (n=23)	11 (47.8)	12 (52.2)
	BSc degree (n=156)	87 (55.8)	69 (44.2)
	Diploma (n=85)	45 (52.9)	40 (47.1)
**Position at facility**			
	Expert position (n=163)	83 (50.9)	80 (66.1)
	Head position (n=101)	60 (59.4)	41 (33.8)
**Experience**			
	≤3 years (n=61)	27 (18.9)	34 (55.7)
	>4 years (n=203)	116 (81.1)	87 (42.9)

^a^All the percentages were calculated for each sociodemographic category.

### Factors Associated With the Commitment Levels to Use DHIS2 Data for Decision Making

PMT members who received feedback for their DHIS2 data use were 1.85 times (adjusted odds ratio [AOR] 1.85, 95% CI 1.02-3.33) more likely to have a higher commitment level to use DHIS2 data than those who did not receive feedback. PMT members who had regular supervision and managerial support on their daily use of DHIS2 data for decision making were 2.84 times (AOR 2.84, 95% CI 1.50-5.37) more likely to have higher levels of commitment to use DHIS2 data than those who had no supportive supervision. Respondents who were competent to use DHIS2 data for their decision making were 2.41 times (AOR 2.41, 95% CI 1.27-4.55) more likely to have higher levels of commitment to use DHIS2 data than those who were not competent in DHIS2 tasks. PMT members with good culture of information use were 1.92 times (AOR 1.92, 95% CI 1.03-3.59) more likely to have higher levels of commitment to use DHIS2 data for decision making than those who did not have good culture of information use. Similarly, PMT members who inquired for DHIS2 data for health management were 3.96 times (AOR 3.96, 95% CI 2.11-7.41) more likely committed to use DHIS2 data than those who did not need DHIS2 data for health management. PMT members having motivation to use DHIS2 data were 1.80 times (AOR 1.80, 95% CI 1.00-3.25) more likely committed to using DHIS2 data when compared to those who had low motivation to use DHIS2 data for their decision making. These data are presented in Table 3.

**Table 3:** Factors associated with the level of commitment to use district health information system 2 data among performance monitoring team members at health facilities in the Ilu Aba Bora zone, Oromia region in 2020^a^.

Variable, category		Commitment level		Crude odds ratio	Adjusted odds ratio
		High commitment, n (%)	Low commitment, n (%)		
**Culture of information use**					
	Good (n=164)	83 (50.6)	81 (49.4)	1.67 (1.00-2.77)*	1.92 (1.03-3.59) **
	Poor (n=100)	38 (38.0)	62 (62.7)	1^b^	1
**Health needs**					
	Yes (n=131)	76 (58.0)	55 (42.0)	2.70 (1.64-4.45)	3.96 (2.11-7.41)***
	No (n=133)	45 (33.8)	88 (66.2)	1	1
**Motivation**					
	High motivation (n=136)	71 (52.2)	65 (47.8)	1.70 (1.04-2.77) *	1.80 (1.00-3.25)**
	Poor motivation (n=128)	50 (39.1)	78 (60.9)	1	1
**Feedback**					
	Yes (n=140)	71 (50.7)	69 (49.3)	1.52 (0.93-2.48)	1.85 (1.02-3.33)**
	No (n=124)	50 (40.3)	74 (59.7)	1	1
**Supervision**					
	Yes (n=141)	84 (59.6)	57 (40.4)	3.42 (2.05-5.71)	2.84 (1.50-5.37)***
	No (n=123)	37 (30.1)	86 (69.9)	1	1
**Competency**					
	High (n=133)	76 (57.1)	57 (42.9)	2.54 (1.54-4.19)	2.41 (1.27-4.55)**
	Low (n=131)	45 (34.4)	86 (65.6)	1	1

^a^All the percentages were calculated for each sociodemographic category.

^b^Reference.

*P<.05 for bivariable analysis.

**P<.05 for multivariable analysis.

***P≤.001.

### Qualitative Results

Interview questions were expected to be directed toward 3 categories of investigation: level of commitment to use DHIS2 data for decision making, factors that could facilitate level of commitment, and challenges to use DHIS2 data for decision making. Analysis of the interview transcripts revealed key themes grouped into one of the above 3 categories. Most of the interviewees agreed that they were able to use DHIS2 data, that they were competent, and that they devoted their time, resources, and efforts to use DHIS2 data.

…Having taken training and also under supervision from my managers, I search DHIS2 data on where and when to do our activities. So I have confidence to say that I am familiar with effective utilization of DHIS2 data for decision making.

HMIS Officer, 27 years old

Respondents said that promoting the culture of information use would increase their confidence in using DHIS2 data.

*…Thereis a good culture for using information. This enables us to carry out our attention to use effectively DHIS data. For this, we are able to compute with technology that inquires oneself to update himself with DHIS2 data used for decision making*. [Medical Director, 29 years old]

Another respondent explained the members’ commitment to use DHIS2 data as follows:

…The PMT members are those who raise why and how questions to make effective use of data for decision making. As a manager of the health center, I’m also playing a role even more than what is expected of me. We are always ready to cut off the problems encountered with using DHIS2 data for decision making. Even we are in need that always like to be guided by DHIS2 data.

TB focal nurse, 30 years old

In some areas, health care providers showed low responsibility toward using DHIS2 data for decision making.

…Some are unresponsive to what they are required to do, some are unaccountable to their duty. We are also facing a lack of budget to use DHIS2 data for decision making. On behalf of the facility, we do not have much materials like computers, internet connections, Wi-Fi, adequately trained human resources.

Triage Head nurse, 26 years old

To achieve a high level of commitment, respondents had problems as follows:

…On behalf of our facility, we have encountered numerous problems such as insufficient computers, no sufficient internet access, and no sufficient trained human power. All of the above use DHIS2 data for decision making at an optimum stage in our facility and we are expected to do more in future.

HMIS officer, 31 years old

…Sometimes there is incomplete data. Sometimes there is too late data. This is due to misunderstanding about using DHIS2 data. Resource is not provided at required stages. Example, we will be out of internet connection for three weeks, our computer may fail but may not be fixed until one month. We are asked to be supported but no response.

MCH Head, 29 years old

## Discussion

This study focused on the level of commitment of health care providers to use DHIS2 data and the factors that affect their levels of commitment. We found that the 45.8% (121/264, 95% CI 40.0%-52.8%) of the PMT members used DHIS2 data for decision making, which was higher than that reported in a study conducted in Iran (35.75%) [19]. This finding may be attributed to the fact that the government of Ethiopia has given special attention to the utilization of health information systems for decision making and the internal commitment of health care providers in Ethiopia to use these data has increased [20]. However, the proportion of PMT members committed to using DHIS2 data in this study was lower than that reported in a study conducted in Ghana (77.3%) [21] and Iran (74.7%) [22]. This might be because infrastructures and advancements in technology in Ghana are more developed than those in Ethiopia. The proportion of the committed PMT members in this study was also lower than that of the PMT members in a study conducted in Nigeria, wherein the proportion of professionals committed to use the routine health information system was 60%-80% [23]; however, the target for this proportion in 2010 was 90% [24]. The possible explanations for this variation could be the size of the study participants, their scope of roles, availability of infrastructure, and availability of resources such as internet connection and other related electronic devices. This result was supported by qualitative findings as follows:

…We familiarized ourselves with DHIS2 data even more than expected from us. We are dedicated to accepting and using DHIS2 data, those who were taken by training everywhere else have given training to those who have not been taken. However we lack some requirements like sufficient internet connection and skills to amend our tools like computers, internet related materials.

Primary health care unit manager, 31 years old

…Almost by what we have, we sacrificed our efforts to use DHIS2 data for our decision making though we encounter some difficulties from the resources limitation.

HMIS officer, 29 years old

PMT members competent in DHIS2 data tasks were 2.41 times more likely to have a higher level of commitment to use DHIS2 data for decision making than those incompetent in DHIS2 data tasks (AOR 2.41, 95% CI 1.27-4.55). This finding was in line with those reported in studies conducted in Ethiopia [25], Ghana [2], Nairobi, Kenya [26], and another study conducted at the health facilities in Kenya (*P*=.03) (AOR 4.32, 95% CI 2.34-7.98) [27]. However, this finding was inconsistent with that of a study conducted in Kenya, which indicated that competency in DHIS2 task has no association with the performance of the health information systems [28]. This result was supported by qualitative finding as follows:

…We ought to have sufficient competency to use DHIS2 data, even we have a good competency in using DHIS2 data tasks though we don’t have enough internet access and sufficient computer devices.

TB focal nurse, 30 years old

This study revealed that feedback on DHIS2 data use was positively associated with PMT members’ commitment level to use DHIS2 data for their decision making in the Ilu Aba Bora zone health facilities (AOR 1.85, 95% CI 1.02-3.33), which was in line with the findings of the studies conducted in Ethiopia [12], Kenya [29], and Ghana (*P*=.04) [2]. However, this finding was inconsistent with the findings of a study conducted in Ghana [30].

The promotion of information use culture in health care providers would result in them being 1.92 times more likely to have higher levels of commitment to use DHIS2 data as compared to those who did not have a culture of information use (AOR 1.92, 95% CI 1.03-3.59). This result was supported by qualitative findings as follows:

…We need to use DHIS2 data for clinical decision making that it enables us to perform our duty more quickly and with full evidence.

Psychiatry Head, 27 years old

As this study revealed, commitment levels to use DHIS2 data for decision making were based on health needs (AOR 3.96, 95% CI 2.11-7.41). However, this finding was inconsistent with that reported in a cross-sectional study conducted in Ghana, which showed that the commitment to use DHIS2 data for decision making does not depend on the health needs [2]. This result was supported by a qualitative finding as follows:

…Applying and using of DHIS2 data for decision making could be tied to health needs, because it is when there is health needs that DHIS2 data will be put in to considerations that it helps us to deal with our focuses.

Triage Head focal nurse, 32 years old

Regarding study participants’ motivation to use DHIS2 data, respondents with higher motivation were 1.80 times more likely to have higher levels of commitment when compared to those with lower motivation to use DHIS2 data for their decision making (AOR 1.80, 95% CI 1.00-3.25). This finding (*P*=.03) was in line with the findings of studies conducted in Ethiopia [25] and Ghana (*P*=.01) [2].

PMT members with regular supportive supervision visits were 2.84 times more likely to have a higher level of commitment than those who did not have regular supportive supervision (AOR 2.84, 95% CI 1.50-5.37). This result was similar to those reported in studies conducted in Ethiopia [12,25] and Ghana, which showed that the level of commitment to use DHIS2 data was directly associated with the daily managerial supervision (*P*=.04) [2].

This study attempted to reveal the commitment levels of health care providers to use DHIS2 data and the factors associated with their levels of commitment. The strength of this study lies in the attempt to cover the different types of health facilities such as health centers, primary hospitals, and referral hospitals. Moreover, our study used a mixed-methods approach and gives evidence on the commitment levels of PMT members to use DHIS2 data for decision making and the barriers in using it. However, our study has the following limitations. First, this study was a facility-based cross-sectional study; therefore, it could not provide the causal relationships with the factors. Second, this study was conducted at health facilities and might not be generalizable to all other administrative services in Ethiopia. In addition, this study did not include health care providers in private health care facilities.

In conclusion, less than half of the PMT members in this study were committed to using DHIS2 data for decision making. Based on WHO’s criteria for commitment to use health information and other studies found in the literatures, our proportion was low. The culture of information use, motivation to use DHIS2 data, competency in DHIS2 tasks, health needs, managerial supervision, and feedback on DHIS2 data use were the most important factors determining the commitment of health care providers to use DHIS2 data for decision making. Thus, we found significant factors that affect PMT members’ level of commitment to the use of DHIS2 data for their decision making. The findings of our study suggest that providing regular supportive supervision and feedback, increasing the motivation of health care providers, and changing their attitudes will help in bringing cultural transformation of data use for evidence-based decision making in health care.

## Abbreviations

AOR: adjusted odds ratio
DHIS2: district health information system 2
HMIS: health management information system
MCH: maternal and child health
PMT: performance monitoring team
TB: tuberculosis
WHO: World Health Organization
